# Gastrointestinal manifestations of *Talaromyces marneffei* infection in an HIV-infected patient rapidly verified by metagenomic next-generation sequencing: a case report

**DOI:** 10.1186/s12879-021-06063-1

**Published:** 2021-04-21

**Authors:** Ying Zhou, Yongfeng Liu, Ying Wen

**Affiliations:** 1 Department of Infectious Diseases, The First Affiliated Hospital of China Medical University, No. 155, Nanjing North Street, Heping District, Shenyang, 110001 Liaoning Province China; 2 BGI PathoGenesis Pharmaceutical Technology, BGI-Shenzhen, Shenzhen, Guangdong Province China

**Keywords:** Human immunodeficiency virus, *Talaromyces marneffei*, Gastrointestinal involvement, Metagenomic next-generation sequencing

## Abstract

**Background:**

The manifestation of *Talaromyces marneffei* infection in some HIV-infected patients may be atypical. Cases with gastrointestinal involvement have rarely been reported. It is hard to make a diagnosis when patients are lacking the characteristic rash and positive blood culture.

**Case presentation:**

Here, we described a patient living with HIV who complained of fever and abdominal pain, and was rapidly diagnosed with *Talaromyces marneffei* infection by metagenomic next-generation sequencing (mNGS) using formalin-fixation and paraffin-embedded (FFPE) samples of omentum majus tissue. We also reviewed reported related cases.

**Conclusions:**

*Talaromyces marneffei* is an unusual cause of clinical presentations involving obvious abdominal pain and lower gastrointestinal bleeding, but can be included in the differential diagnosis. As an important diagnostic tool, the significance of mNGS using FFPE samples of lesions provides a more targeted diagnosis.

## Background

The common manifestations of *Talaromyces marneffei* infection in human immunodeficiency virus (HIV)-infected individuals consist of fever, anemia, weight loss, characteristic skin papules, respiratory signs, lymphadenosis, hepatosplenomegaly, and other organ involvement. In China, *Talaromyces marneffei* is mainly found in southern China. Therefore, HIV-infected patients with a travel history to southern China should have *Talaromyces marneffei* infection considered when they first present with gastrointestinal manifestations.

## Case presentation

A 33-year-old Chinese man presented with continuous fever for one month from July 15th, 2019, followed by 20 days of abdominal pain. The initial highest temperature was 38.9 °C, accompanied by night sweats, anorexia, fatigue, weight loss and diarrhea (watery stool, 4–5 times per day). On July 25th, 2019, the patient presented with intolerable abdominal pain and body temperature had increased to 40 °C. HIV infection was confirmed and the patient’s CD4^+^ T-cell count was 7cells/μL. The patient was born in Shenyang, located in the north of China. However, since July 2018, he had worked and travelled a lot in Guangdong province, located in the south of China. The patient had eaten roasted bamboo rat in December 2018.

**Fig. 1:**
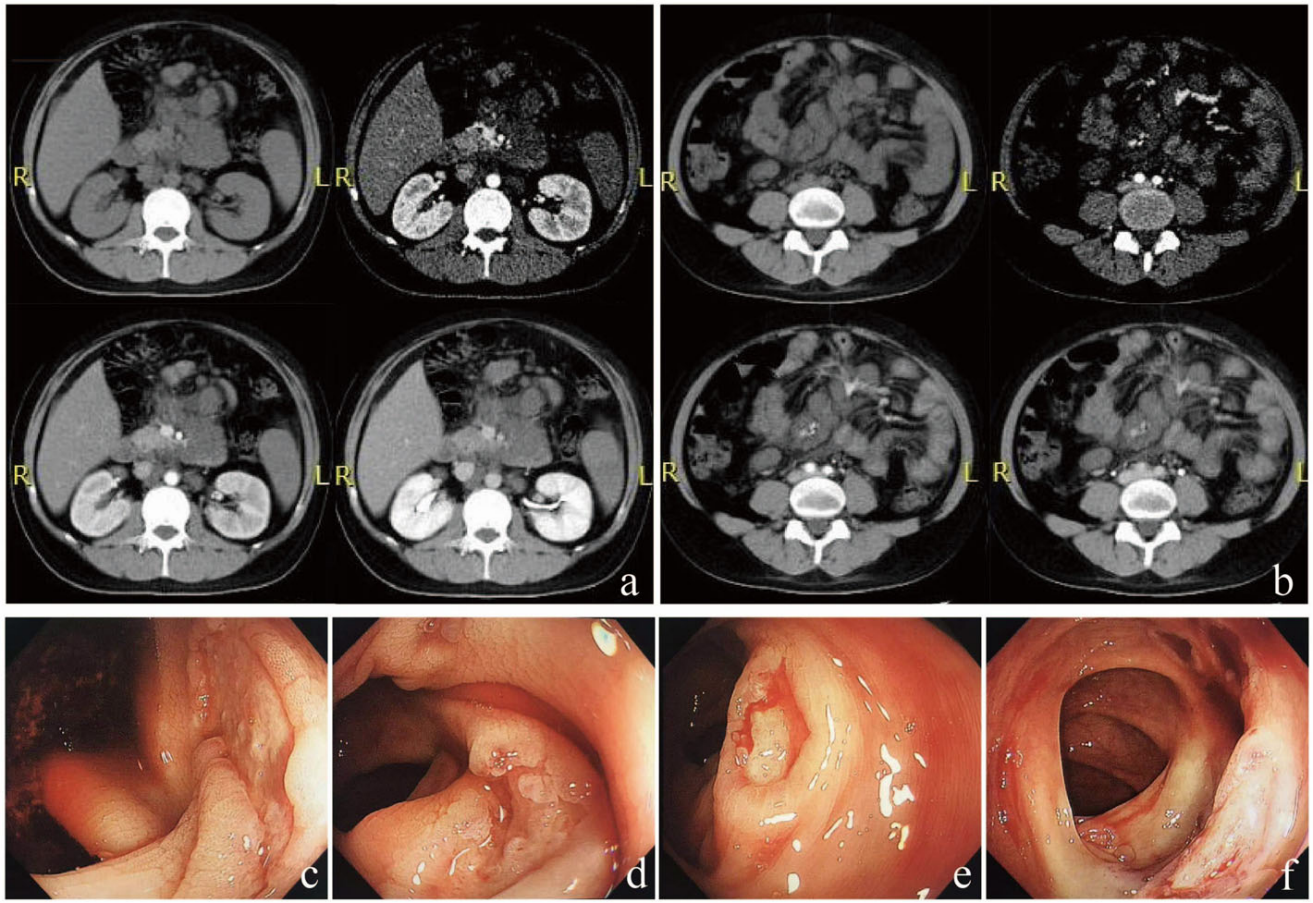
Presentation of abdominal CT scan and colonoscopy. Abdominal CT scan showed severe fatty liver, pelvic cavity effusion (**a**), thickened and swollen small intestinal wall and thickened mesentery, accompanied by multiple enlarged intra-abdominal lymph nodes (**a**, **b**). Gastrointestinal endoscopy found multiple small shallow ulcers scattered in the cecum (**c**), ascending colon (**d**), transverse colon (**e**), and descending colon (**f**), partly accompanied by white exudates and active bleeding

The patient experienced abdominal tenderness and rebound pain. Brain contrast magnetic resonance imaging (MRI) and chest computed tomography (CT) scans were relatively normal. Abdominal CT scans showed severe fatty liver, thickened and swollen small intestinal wall, pelvic cavity effusion, and thickened mesentery accompanied by multiple enlarged intra-abdominal lymph nodes (Fig. 1a, b). The patient had normal leucocyte and platelet counts and mild anemia. The patient displayed elevated C-reactive protein (135.30 mg/L) and galactomannan levels (4.39 μg/L). Serum ferritin was above 2000.00 μg/L. Serum cryptococcal antigen was negative. Anti-neutrophil cytoplasmic antibodies were negative. Toxoplasma gondii IgM and IgG antibodies were negative. Cytomegalovirus (CMV) IgG antibody and herpes simplex virus IgG antibody were positive, while IgM antibodies were negative. Epstein-Barr virus (EBV) IgM antibody was negative while viral capsid antigen IgG and nuclear antigen IgG antibodies were positive. Serum CMV-DNA was undetectable. Whole blood EBV-DNA was undetectable. The HIV RNA load was 5.1 × 10^5^ copies/mL. Blood bacteria and fungi cultures were repeated three times and all tests were negative. Fecal bacteria and fungi cultures were repeated three times and all tests were negative. We did not observe parasite eggs in the stool specimens. Giemsa stain of bone marrow aspirate did not find any pathogens. A bone marrow sample culture was not carried out due to insufficient bone marrow samples obtained.

**Fig. 2:**
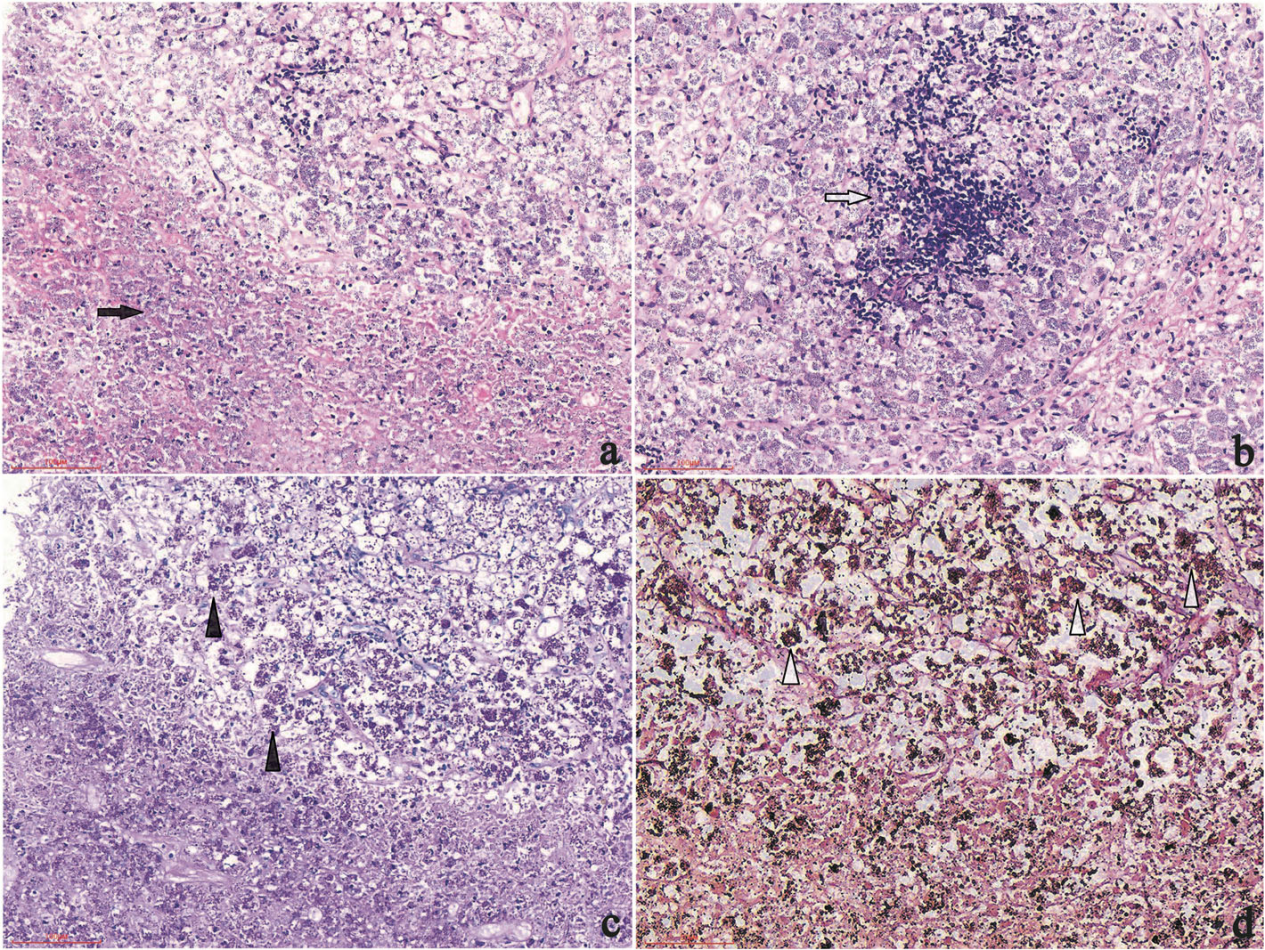
Histopathology of biopsy samples. H&E staining showing granuloma with central necrosis and concentrated inflammatory cell infiltrations involving foamy macrophages (containing a large number of yeasts), neutrophils (**a**) (200 × magnification), and lymphocytes (**b**) (200 × magnification); Yeasts with positive PAS staining (**c**) (200 × magnification) and GMS staining (d) (400 × magnification) in macrophages

**Fig. 3:**
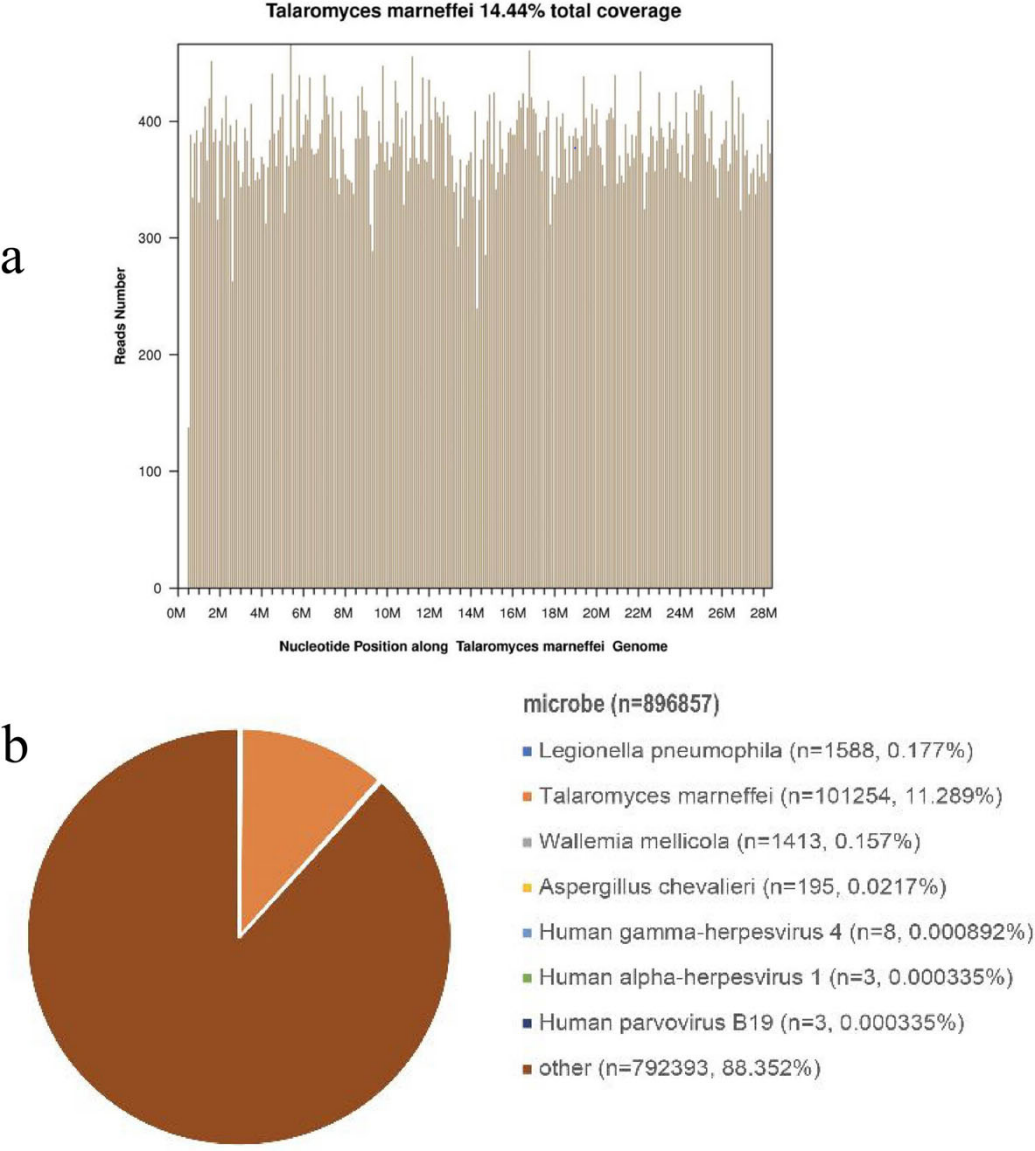
Pathogen identification from paraffin-embedded tissue samples using the metagenomic next-generation sequencing method. The number of sequencing reads identified that corresponded to *Talaromyces marneffei* was 101,254 (**b**) with 14.44% genome coverage (**a**). Reads distribution of the total DNA sequence in the sample was without human host

For this kind of patient with obvious peritonitis despite a negative T-SPOT result, empirical anti-tuberculosis treatment with a regimen of isoniazid, rifampin, ethambutol, and pyrazinamide was prescribed at the day 4 of admission. A week later, fever and abdominal pain had worsened. The patient complained of diffuse abdominal pain and sustained fever. The patient displayed abdominal rigidity. At the day 12 of admission, fungal infection was suspected and omentum majus biopsy was performed. Hematoxylin and eosin (H&E) staining showed granuloma with central necrosis and a large number of foamy macrophages, lymphocytes, and neutrophil infiltration. Periodic acid–Schiff (PAS) and Gomori’s methenamine silver nitrate (GMS) staining showed clustered yeast in macrophages (Fig. 2). Acid-fast bacilli staining (using Ziehl Neelsen), CMV-antigen, TB-DNA, and EBV-DNA in paraffin-embedded tissue sections were all negative. The patient began antifungal treatment with amphotericin B. In order to identify the specific fungal species, particularly to differentiate between *Talaromyces marneffei*, histoplasma, and other deep fungal infections, FFPE samples were sent to BGI PathoGenesis Pharmaceutical Technology (BGI-Shenzhen) for metagenomic next-generation sequencing (mNGS), which indicated *Talaromyces marneffei* infection 3 days later (Fig. 3). In brief, the experimental procedure was performed as follows: DNA from the patient’s FFPE samples was extracted using the MagPure FFPE DNA LQ Kit following the manufacturer’s instructions. The DNA library was constructed and sequenced, human sequences were excluded, and low-complexity reads were removed, the remaining data were classified by simultaneously aligning to four microbial genome databases, consisting of 4061 whole genome sequences of viral taxa, 2473 bacterial genomes or scaffolds, 199 fungi connected to human infection, and 135 parasites associated with human diseases [1].

Fever and abdominal pain rapidly resolved after the initial days of starting amphotericin B treatment, while gastrointestinal bleeding occurred with a total bloody stool volume of 1000 mL. Gastrointestinal endoscopy revealed multiple small shallow ulcers scattered in the cecum, ascending colon, transverse colon, and descending colon, partly accompanied by white exudates and active bleeding (Fig. 1c-f). After endoscopic hemostasis therapy with 1:1 epinephrine solution injected around the lesion, the bleeding temporarily stopped. Amphotericin B treatment was continued followed by oral itraconazole. Intestinal bleeding had another two relapses and achieved spontaneous remission. The patient’s serum creatinine increased to 120 μmol/L during amphotericin B treatment, but tenofovir alafenamide fumarate was not available in China at that time, therefore, we suggested to use abacavir, and spent weeks to detect HLA-B5701 and applied for abacavir. After eight weeks of anti-fungal treatment, ART was initiated with a regimen of lamivudine, abacavir, and dolutegravir. After 12 weeks of anti-fungal treatment, abdominal CT indicated that the thickened mesentery and small intestine had recovered, the retroperitoneal lymph node had shrunk, and colonoscopy showed that the colon lesions had recovered. A 12-month follow-up revealed that the patient’s CD4+ T-cell count had increased to 85cells/μL and HIV RNA was undetectable. The patient continues to take 200 mg itraconazole per day as secondary prevention until CD4 + T cells count reach 100 cells/μL for at least 6 months.

## Discussion and conclusions

This is a case of gastrointestinal *Talaromyces marneffei* infection with negative blood culture, and the absence of any respiratory involvement or rash. The mNGS rapidly aided in identifying *Talaromyces marneffei* nucleotide sequences in omentum majus FFPE samples from our patient, which had never been previously reported; In 3 previous published papers, mNGS has been reported to help to diagnose *Talaromyces marneffei* infection in the bronchoalveolar lavage fluid [2, 3], bone marrow [2], cerebrospinal fluid [2, 4], and skin lesion [2] specimens.

*Talaromyces marneffei* is a common opportunistic infection among HIV-infected patients in southeast Asia, southern China, and northeastern India, which are endemic areas for *Talaromyces marneffei.* Possible epidemiological risk factors are as follows: (1) a history of travel or living in endemic areas and soil exposure, especially during the rainy season, has been suggested to be a critical risk factor; (2) people living with HIV infection, especially CD4^+^ T-cell counts below 200cells/μL, contributes to an increased risk of *Talaromyces marneffei* infection. Common manifestations of disseminated *Talaromyces marneffei* include fever, anemia, weight loss, skin lesions, respiratory signs, lymphadenopathy, and hepatosplenomegaly. Characteristic cutaneous lesions aids to diagnosis and *Talaromyces marneffei* infection can be confirmed by positive culture from blood, skin lesion, and bone marrow samples [5]. Inhalation of conidia is the primary route of infection, which then disseminates to the reticuloendothelial system, skin, and gastrointestinal organs. Although gastrointestinal symptoms (e.g., diarrhea) are relatively common with a prevalence of approximately 25% [6], the prevalence of colonic involvement caused by *Talaromyces marneffei* infection is only 1.9% [7]. Including the present case, prominent abdominal involvement from *Talaromyces marneffei* infection has been reported in a total of 14 patients (Table 1) [4–13]. The main macroscopic pathological changes include multiple gastrointestinal ulcers and mesenteric lymphadenitis. Common distribution of colonic infections include the cecum, ascending colon, appendix, transverse colon, descending colon, or sigmoid colon, small intestine, and duodenum. Common clinical manifestations are fever, diarrhea, abdominal pain, lower gastrointestinal bleeding, and intestinal obstruction. Most patients survive with anti-fungal treatment. Wild bamboo rats exhibit a 100% prevalence of *Talaromyces marneffei* infection [14]. It is important to note that the bamboo rat is a common species of rodent bred for meat and wool in southern China. The potential for bamboo rats to transmit pathogens to humans remains unclear because most patients with *Talaromyces marneffei* infection in Guangdong did not have a history of contact with bamboo rats [15]. Although the patient’s history of bamboo rat consumption is very suggestive, the link between bamboo rat ingestion history in this case and predominantly gastrointestinal presentation requires further study.

**Table 1:** Summary of clinical characteristics for 14 HIV-infected cases with intestinal *Talaromyces marneffei*

Case No.	Age (yr)/gender	Area and year of report	Abdominal symptoms	Other clinical presentations	Skin and mucous membrane appearance	Involved organ or tissue/diagnostic methods	Treatment maintenance	Outcome
1	72/M	Hong Kong China 1992 [4]	GI bleeding	anorex*i*a, dysphagia, weight loss	jejunal ulcer(S)	small intestine(B + C), mesenteric lymph node, liver(A)	NM	Died
2	32/M	Hong Kong China 1996 [5]	diarrhea	fever, night sweats, dry cough	multiple solitary ulcers(E)	cecum, transverse and descending colon(B + C)	Amphotericin B/Itraconazole	survived
3、4	NM	Thai 1998 [8]	abdominal pain	fever	NM	mesenteric lymph node (B), blood and bone marrow (C)	Amphotericin B	survived
5	52/M	Taiwan China 1999 [6]	diarrhea, abdominal pain	fever, erupted papule, anomia,	shallow ulcers(E)	skin, bone marrow(B + C), colons(B)	Amphotericin B/Itraconazole	survived
6	30/M	Taiwan China 1999 [6]	diarrhea, abdominal pain, bloody stool	dyspepsia, fever, anomia, weight loss	shallow ulcers(E)	cecum, ascending and transverse colons(B + C)	Amphotericin B/Itraconazole	survived
7	33/M	India 2008 [7]	abdominal pain	fever, loss of appetite, weight loss, vomiting	duodenum narrowing(E)	duodenum(B + C), bone marrow(C)	Amphotericin B/Itraconazole	survived
8	39/M	Hong Kong China 2010 [9]	Abdominal pain	fever, weight loss	perioral umbilicated lesions	neck and retroperitoneal lymph nodes (H + C),blood (C)	Amphotericin B/Itraconazole	Survived
9	28/M	India 2014 [10]	non-colicky abdominal pain	fever, weight loss	perioral umbilicated lesions	neck nodes and retroperitoneal lymph nodes(B + C), blood(C)	Amphotericin B/Itraconazole	survived
10	52/M	China 2017 [11]	pain in the lower left abdomen	anorexia, weight loss	multiple solitary shallow ulcers (E)	transverse colon (B + H)	Itraconazole	survived
11	38/F	India 2020 [12]	colicky abdominal pain	loss of appetite, weight loss	skin lesions, jejunal ulcers(E)	skin, jejunal ulcers(B + C),	Amphotericin B/Itraconazole	survived
12	37/M	China 2020 [13]	Abdominal pain	NM	multiple ulcers (E)	colon (B), blood (C)	Amphotericin B/Itraconazole	Survived
13	50/M	China 2020 [13]	Abdominal pain	weight loss	multiple ulcers (E)	colon (B)	Voriconazole+Amphotericin B/Itraconazole	Survived
14	33/M	China [PR]	colicky abdominal pain, bloody stool	fever, weight loss, night sweats	colon ulcers(E)	Mesenteric lymph node(B + N)	Amphotericin B/Itraconazole	survived

*ND* Not done, *NM* not mentioned, *PR* present report

Diagnostic methods to demonstrate P marneffei were autopsy (A), biopsy (B), culture (C), histopathology (H), surgery(S), Endoscope(E), NGS(N)

This case report has several limitations. The limited size of the omentum majus biopsy tissue was insufficient for tissue culture. Another limitation is a lack of microbial cultivation of bone marrow aspirate. It is unfortunate that we did not perform biopsies of the ulcers identified during the endoscopic examination due to intolerance of the patient and the risk of hemorrhage. Although the lack of an intestinal pathological confirmation from non-specific shallow ulcers infiltrated by lymphocytes and histiocytes distended with yeast [6], intestinal *Talaromyces marneffei* infection was considered based on the patient’s abdominal symptoms of diarrhea, abdominal pain, and bloody stool, which showed total improvement following anti-fungal treatment.

In conclusion, as a type of culture-independent method, mNGS provides a rapid etiological diagnosis, especially in patients with an uncommon presentation of *Talaromyces marneffei* infection. FFPE samples of lesions and fresh biopsy specimens may represent suitable specimens for mNGS, which may be convincing for obtaining a targeted diagnosis and treatment.

## Data Availability

Not applicable (no datasets were generated or analyzed during the current report).

## Abbreviations

HIV: Human immunodeficiency virus
mNGS: Metagenomic next-generation sequencing
MRI: Magnetic resonance imaging
CT: Computed tomography
H&E: Hematoxylin and eosin
PAS: Periodic acid–Schiff
GMS: Gomori’s methanamine silver nitrate staining
FFPE: Formalin-fixation and paraffin-embedded

## References

[1] Zhu YM, Ai JW, Xu B, Cui P, Cheng Q, Wu H, Qian YY, Zhang HC, Zhou X, Xing L, Wu R, Li Y, Zhang WH (2018). Rapid and precise diagnosis of disseminated T.marneffei infection assisted by high-throughput sequencing of multifarious specimens in a HIV-negative patient: a case report. BMC Infect Dis.

[2] Pongpech N, Rotjanapan P (2019). Absence of cutaneous involvement in disseminated Talaromyces marneffei infection in an AIDS patient: a case report and literature review. Infect Drug Resist.

[3] Praneenararat S (2014). Fungal infection of the colon. Clin Exp astroenterol.

[4] Tsui WMS, Ma KF, Tsang DNC (1992). Disseminated Penicillium marneffei infection in HIV-infected subject. Histopathology..

[5] Leung R, Sung JY, Chow J, Lai CKW (1996). Unusual cause of fever and diarrhea in a patient with AIDS: Penicillium marneffei infection. Dig Dis Sci.

[6] Ko CI, Hung CC, Chen MY, Hsueh PR, Hsiao CH, Wong JM (1999). Endoscopic diagnosis of intestinal penicilliosis marneffei: report of three cases and review of the literature. Gastrointest Endosc.

[7] George IA, Sudarsanam TD, Pulimood AB, Mathews MS (2008). Acute abdomen: an unusual presentation of disseminated Penicillium marneffei infection. Indian J Med Microbiol.

[8] Ukarapol N, Sirisanthana V, Wongsawasdi L. Penicillium marneffei mesenteric lymphadenitis in human immunodeficiency virus-infected children. J Med Assoc Thail. 1998;81(8):637–40. 9737118

[9] Hung HG, Lok KH. Intestinal Penicillium marneffei: an unusual cause of chronic diarrhea in an AIDS patient. J Dig Dis. 2010;11(3):189–91. 10.1111/j.1751-2980.2010.00435.x. 10.1111/j.1751-2980.2010.00435.x 20579223

[10] Ghalige HS, Sahoo B, Sharma S, Devi KR, Singh Th SC. Acute abdomen due to Penicillium marneffei: an Indicator of HIV infection in Manipur state. J Clin Diagn Res. 2014;8(9):ND05–6. 10.7860/JCDR/2014/9632.4825 PMC4225934 25386482

[11] Feng S, Wang X, Zhang X, Yang H, Wang Z. Pathological diagnosis of a rare intestinal penicillium marneffei infection in an acquired immunodeficiency syndrome patient: a case report and literature review. Int J Clin Exp Pathol. 2017;10(3):3710–5.

[12] Philip Sridhar R, Coelho VV, Roopavathana B, Chase S. Opportunistic penicilliosis infection causing intestinal obstruction in people living with HIV complicating antiretroviral therapy. BMJ Case Rep. 2020;13(2):e230121. 10.1136/bcr-2019-230121 PMC7046417 32060105

[13] Pan M, Huang J, Qiu Y, Zeng W, Li Z, Tang S, Wei X, Zhang J. Assessment of Talaromyces Marneffei Infection of the Intestine in Three Patients and a Systematic Review of Case Reports. Open Forum Infect Dis. 2020;7(6):ofaa128. 10.1093/ofid/ofaa128 PMC7264840 32523970

[14] Cao C, Liang L, Wang W, Luo H, Huang S, Liu D, et al. Common reservoirs for Penicillium marneffei infection in humans and rodents. China Emerg Infect Dis. 2011;17(2):209–14. 10.3201/eid1702.100718. 10.3201/eid1702.100718 PMC3204759 21291590

[15] Li X, Yang Y, Zhang X, Zhou X, Lu S, Ma L, et al. Isolation of Penicillium marneffei from soil and wild rodents in Guangdong. SE China Mycopathologia. 2011;172(6):447–51. 10.1007/s11046-011-9443-5. 10.1007/s11046-011-9443-5 21744044

